# *p-Coumaric* Acid Influenced Cucumber Rhizosphere Soil Microbial Communities and the Growth of *Fusarium oxysporum* f.sp. *cucumerinum* Owen

**DOI:** 10.1371/journal.pone.0048288

**Published:** 2012-10-30

**Authors:** Xingang Zhou, Fengzhi Wu

**Affiliations:** Department of Horticulture, Northeast Agricultural University, Xiangfang, Harbin, People’s Republic of China; Argonne National Laboratory, United States of America

## Abstract

**Background:**

Autotoxicity of cucumber root exudates or decaying residues may be the cause of the soil sickness of cucumber. However, how autotoxins affect soil microbial communities is not yet fully understood.

**Methodology/Principal Findings:**

The aims of this study were to study the effects of an artificially applied autotoxin of cucumber, *p*-coumaric acid, on cucumber seedling growth, rhizosphere soil microbial communities, and *Fusarium oxysporum* f.sp. *cucumerinum* Owen (a soil-borne pathogen of cucumber) growth. Abundance, structure and composition of rhizosphere bacterial and fungal communities were analyzed with real-time PCR, PCR-denaturing gradient gel electrophoresis (DGGE) and clone library methods. Soil dehydrogenase activity and microbial biomass C (MBC) were determined to indicate the activity and size of the soil microflora. Results showed that *p*-coumaric acid (0.1–1.0 µmol/g soil) decreased cucumber leaf area, and increased soil dehydrogenase activity, MBC and rhizosphere bacterial and fungal community abundances. *p*-Coumaric acid also changed the structure and composition of rhizosphere bacterial and fungal communities, with increases in the relative abundances of bacterial taxa *Firmicutes*, *Betaproteobacteria*, *Gammaproteobacteria* and fungal taxa *Sordariomycete*, *Zygomycota*, and decreases in the relative abundances of bacterial taxa *Bacteroidetes*, *Deltaproteobacteria*, *Planctomycetes*, *Verrucomicrobia* and fungal taxon *Pezizomycete*. In addition, *p*-coumaric acid increased *Fusarium oxysporum* population densities in soil.

**Conclusions/Significance:**

These results indicate that *p*-coumaric acid may play a role in the autotoxicity of cucumber via influencing soil microbial communities.

## Introduction

Soil sickness is a reduction in both crop yield and quality caused by continuous mono-cropping in the same land. It is one of the major problems in agricultural production, especially for greenhouse crops [1]. Cucumber (*Cucumis sativus* L.), a crop of high economic importance in many countries, is vulnerable to soil sickness [1]. Recently, cultivation of cucumber under greenhouse conditions has greatly expanded in China, but significant agricultural loss is observed each year because continuous mono-cropping practice is becoming more and more popular.

The accumulation of autotoxins is probably responsible for the soil sickness of cucumber [1]. Autotoxicity is an intraspecific allelopathy, where a plant species inhibits the growth of plants of the same species through releasing toxic chemicals into the environment [2]. Cucumber root exudates and plant debris were shown to have autotoxicity potential [1]. Autotoxins, including some phenols, have been identified in cucumber root exudates [3]. Some phenols from living and decomposing plant tissues can be active allelochemicals and they can accumulate in soil and have detrimental effects on the growth of associated and next-season plants [4].

Soil microorganisms may influence the persistence, availability and biological activities of allelochemicals in soil [5], [6] and root exudates or allelochemicals can affect soil microbial communities [7]. Thus, it is suggested that allelopathy can be better understood in terms of soil microbial ecology [5], [8]. Recently, a great research effort has addressed the effects of plant root exudates, such as low molecular carbohydrate, organic acids, amino acids, and plant secondary metabolites (e.g. flavonoids and glucosinolate) on soil microbial communities [9]–[13]. Phenols were shown to affect the growth of microorganisms *in vitro* [14], but little information is available on how soil microbial communities respond to putative allelochemicals in natural soils [15], [16].

Our knowledge about effects of autotoxins, such as phenols, on soil microorganism populations has been mainly achieved by traditional cultivation-dependent methods, which is limited in that only a small fraction of the microorganisms are accessible to study [17]–[19]. Analysis of soil microbial communities with molecular techniques, such as real-time PCR, PCR-denaturing gradient gel electrophoresis (DGGE) and phylogenetic analysis, can improve our understanding of how autotoxins affect abundance, structure and composition of soil microbial communities [20], [21].

**Figure 1:**
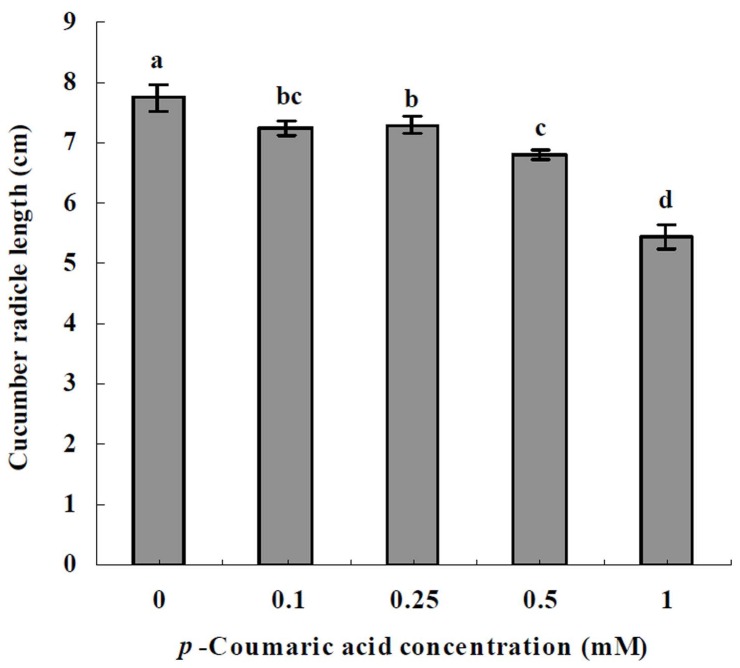
Effects of *p*-coumaric acid on cucumber radicle elongation. Data are represented as the means of three independent replicates with standard error bars. *Different letters* indicate significant difference between treatments (P<0.05, Tukey’s HSD test).

Several studies have reported that soil may become suppressive in the case of long-term monoculture, for example, *Gaeumannomyces graminis* var. *tritici* (take-all disease) of wheat [22], *Rhizoctonia solani* of sugar beet [23], *Thielaviopsis basicola* of tobacco [24] and Fusarium wilt of watermelon [25]. However, it is also suggested that the accumulation of soil-borne pathogens is responsible for the soil sickness [26]–[28]. The *Fusarium* (*Ascomycota*, *Fungi*) community size was found to be linked to the soil sickness associated with cucumber cultivation [28]. Autotoxins would accumulate under continuous mono-cropping conditions; therefore, how autotoxins, such as phenols, affect soil-borne pathogens in soil needs to be further clarified.

**Table 1:** Effects of *p*-coumaric acid on cucumber seedling growth.

Concentration (µmol/g soil)	Leaf area (cm^2^/plant)	Plant height (mm)	Dry weight (mg/plant DW)
0	209±6 a	75±1 a	638±13 a
0.1	179±11 ab	70±3 ab	593±53 ab
0.25	164±9 b	67±3 ab	554±21 ab
0.5	160±7 b	64±4 ab	537±9 ab
1.0	145±8 b	63±3 b	498±9 b
**ANOVA**			
F_4,12_	8.48	3.69	4.01
P-value	0.003	0.043	0.034

Values (mean ± standard error) in the same column followed by different letters are significantly different (P<0.05, Tukey’s HSD test).

*p*-Coumaric acid (*p*-hydroxycinnamic acid) has been identified in plant root exudates or residues [14] and in soils under many plant species, including cucumber [29], [30]. We hypothesized that *p*-coumaric acid could influence cucumber growth and soil microbial communities. The primary aims of this research were to study: 1) the phytotoxic effects of *p*-coumaric acid on cucumber radicle elongation and seedling growth; 2) the effects of *p*-coumaric acid on structure, composition, abundance, activity and size of cucumber rhizosphere microbial communities; 3) the effects of *p*-coumaric acid on the growth of *F. oxysporum* f.sp. *cucumerinum* Owen (a soil-borne pathogen of cucumber) both *in vitro* and in soil. Abundance, structure and composition of bacterial and fungal communities were analyzed by real-time PCR, PCR-DGGE and clone library methods. Soil dehydrogenase activity and microbial biomass C (MBC) were determined to indicate the activity and size of soil microflora.

**Table 2:** Effects of *p*-coumaric acid on soil pH, available N, *p*-coumaric acid in soil, dehydrogenase activity, MBC and bacterial and fungal abundance in cucumber rhizosphere.

Concentration (µmol/g soil)	pH (1∶2.5, w/v)	Available N (mg/kg)	*p*-Coumaric acid retained in soil (µmol/g soil)	Dehydrogenase activity (µg TPF/g soil/24 h)	MBC (mg C/kg soil)	Bacterial abundance (10^11^ copies/g soil)	Fungal abundance (10^8^ copies/g soil)	Bacteria-to-fungi ratio (10^3^)
0	7.72±0.15 a	60.83±4.01 ab	0.14±0.02 d	1.21±0.04 b	152±1 d	4.29±0.10 d	1.30±0.02 d	3.31±0.13 a
0.1	7.94±0.10 a	66.2±1.93 a	0.18±0.02 cd	2.34±0.18 a	199±4 c	7.15±0.42 c	3.72±0.04 c	1.92±0.12 c
0.25	7.77±0.08 a	56.92±2.29 bc	0.28±0.04 c	2.63±0.21 a	220±6 bc	11.25±0.13 a	4.09±0.06 b	2.75±0.06 b
0.5	7.93±0.12 a	58.32±2.42 bc	0.48±0.05 b	2.76±0.13 a	265±5 a	8.87±0.21 b	4.58±0.03 a	1.94±0.04 c
1.0	7.98±0.08 a	52.78±2.47 c	0.87±0.06 a	2.84±0.10 a	235±5 b	8.26±0.11 b	3.74±0.03 c	2.21±0.01 c
**ANOVA**								
F_4,12_	1.09	9.99	173.57	20.25	88.89	123.31	1057.35	48.81
P-value	0.411	0.002	<0.0001	<0.0001	<0.0001	<0.0001	<0.0001	<0.0001

Values (mean ± standard error) in the same column followed by different letters are significantly different (P<0.05, Tukey’s HSD test).

## Materials and Methods

### Cucumber Radicle Elongation Experiment

Ten germinated cucumber seeds (cv. ‘Jinlv 3’) with radicles of 1 mm length were separately placed in a Petri dish (9 cm diameter), which contained two layers of sterilized filter papers. Five milliliter of different concentrations of *p*-coumaric acid solutions (0.1, 0.25, 0.5 or 1.0 mM) with pH adjusted to 7.0 with 0.1 M NaOH solution were added in the Petri dish. Cucumber seeds treated with distilled water (pH 7.0) were used as the control. There were five treatments (four concentrations of *p*-coumaric acid and one control) in total. We incubated three replicates for each treatment, and had five Petri dishes per replicate. The radicle length was measured 10 days after incubation at 25°C in the dark.

**Figure 2:**
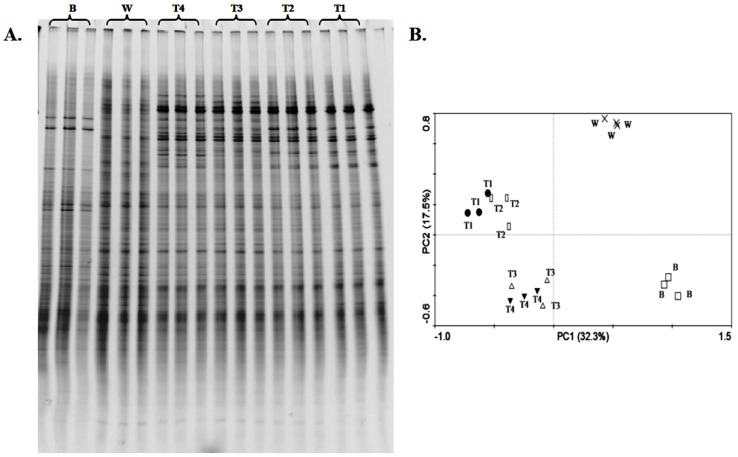
Effects of *p*-coumaric acid on bacterial community structure. (A) DGGE profiles of partial bacterial 16S rRNA gene sequences. (B) Principal component analysis (PCA) of bacterial community based on DGGE profiles. *B* and *W* represent soil sample before cucumber planting and control, respectively. *T1, T2, T3* and *T4* represent soil treated with 0.1, 0.25, 0.5, 1.0 µmol *p*-coumaric acid/g soil, respectively.

**Table 3:** Number of visible bands (*S*), Shannon index (*H*) and evenness index (*E*) based on DGGE analysis of bacterial and fungal communities on DGGE profiles.

*p*-Coumaric acid concentration (µmol/g soil)	Bacterial community			Fungal community		
	*S*	*H*	*E*	*S*	*H*	*E*
B	47±1 ab	3.70±0.04 ab	0.88±0.01 ab	29±3 b	2.96±0.07 a	0.80±0.02 a
0	51±3 a	3.81±0.04 a	0.91±0.01 a	34±1 a	3.09±0.04 a	0.83±0.01 a
0.1	47±2 ab	3.68±0.05 b	0.88±0.01 b	29±1 b	2.98±0.06 a	0.80±0.02 a
0.25	45±1 b	3.66±0.01 b	0.87±0.00 b	31±2 ab	3.00±0.07 a	0.81±0.02 a
0.5	39±2 c	3.47±0.05 c	0.83±0.01 c	33±0 ab	3.09±0.04 a	0.83±0.01 a
1.0	38±2 c	3.45±0.05 c	0.82±0.01 c	32±1 ab	3.04±0.03 a	0.82±0.01 a
**ANOVA**						
F_5,12_	18.89	29.51	29.51	4.88	3.45	3.45
P-value	<0.0001	<0.0001	<0.0001	0.012	0.036	0.036

Values (mean ± standard error) in the same column followed by different letters are significantly different (P<0.05, Tukey’s HSD test).

*B* soil sample before cucumber planting.

### Cucumber Seedling Experiment

Cucumber seedlings with two cotyledons were transplanted into cups (5.5 cm bottom diameter, 8 cm top diameter, 8.5 cm height) containing 250 g soil. The soil was sampled from the upper soil layer (0–15 cm) of an open field in the experimental station of Northeast Agricultural University, Harbin, China (45°41′N, 126°37′E), which was covered with grass and undisturbed for more than 15 years. The soil was a black soil (Mollisol) with sandy loam texture: organic matter, 3.67%; available N, 89.02 mg/kg; available P, 63.36 mg/kg; available K, 119.15 mg/kg; EC (1∶2.5, w:v), 0.33 mS cm^−1^; and pH (1∶2.5, w:v), 7.78. No fertilizer was added to soil. There was one cucumber seedling per cup. The seedlings were incubated in the greenhouse (32°C day/22°C night, relative humidity of 60–80%, 16 h light/8 h dark).

**Figure 3:**
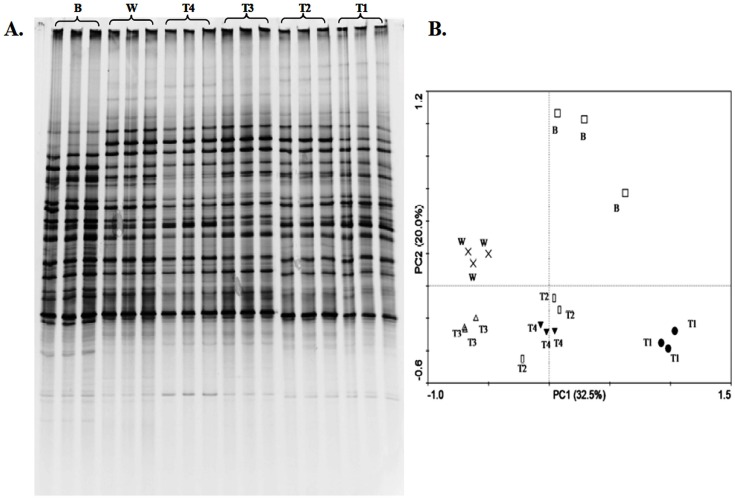
Effects of *p*-coumaric acid on fungal community structure. (A) DGGE profiles of partial fungal ITS regions. (B) Principal component analysis (PCA) of fungal community based on DGGE profiles. *B* and *W* represent soil sample before cucumber planting and control, respectively. *T1, T2, T3* and *T4* represent soil treated with 0.1, 0.25, 0.5, 1.0 µmol *p*-coumaric acid/g soil, respectively.

Phenols can be rapidly depleted after their addition to soil due to microbial degradation [17]. In this study, *p*-coumaric acid was added into soil periodically to maintain the desired concentrations. Our previous study found that *p*-coumaric acid in soils repeatedly cultivated with cucumber ranged from 0.51 to 0.62 µmol/g soil (data not shown). Therefore, cucumber seedlings at the one-leaf stage were treated with different concentrations of *p*-coumaric acid every 48 h to achieve final concentrations of 0.1, 0.25, 0.5, 1.0 µmol/g soil DW. The pH of *p*-coumaric acid solution was adjusted to 7.0 with 0.1 M NaOH solution because soil pH is a dominant factor that regulates soil microbial communities [31]. The soil treated with distilled water (pH 7.0) was used as the control. *p*-Coumaric acid in the soil before cucumber planting (the field soil) was 0.06 µmol/g soil DW, which was considered when adding *p*-coumaric acid: the final concentration of *p*-coumaric acid was the sum of the concentration in the field soil and the concentration added. Soil water content was adjusted every two days with distilled water to maintain a constant weight of cups. There were five treatments (four concentrations of *p*-coumaric acid and one control) in total. We had three replicates for each treatment, and had five seedlings per replicate.

**Figure 4:**
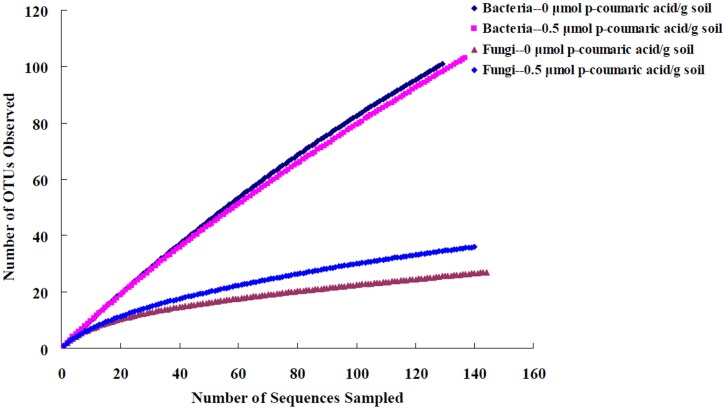
Rarefaction analysis of bacterial 16S rRNA and fungal ITS gene clone libraries. The total number of sequences per library is plotted against the number of unique OTUs encountered within the same library. OTUs were defined at the 97% similarity level.

**Table 4:** Diversity indices for the soil bacterial and fungal communities as represented by clone libraries.

	*p*-Coumaric acid concentration (µmol/g soil)	No of sequences	No of OTUs	Coverage (%)	Chao 1	Shannon (*H*)	Evenness (*E*)
Bacterial community							
	0	129	101	40	259	4.54	0.98
	0.5	137	103	38	400	4.48	0.97
Fungal community							
	0	144	27	90	57	2.62	0.79
	0.5	140	36	86	50	2.78	0.78

Calculations were based on OUTs formed at the 97% similarity level.

Ten days later, cucumber seedlings were destructively sampled and separated into leaves, stems and roots. Five plants per replicate were destructively sampled, and different tissues of the five plants were pooled to make three composite replicates per treatment. Cucumber leaves were scanned with a Microtek ScanMaker i800 plus system (WSeen, China) and leaf area was calculated with a LA-S Leaf Area Analysis software (WSeen, China). Plant dry weight was measured after oven drying at 70°C to constant weight.

**Figure 5:**
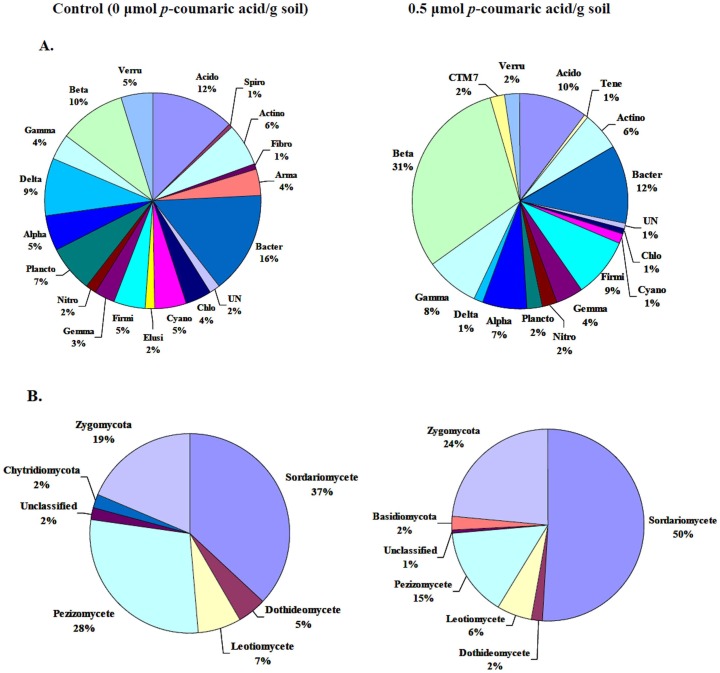
Effects of *p*-coumaric acid on bacterial and fungal community composition. (A) relative abundances of bacterial 16S rRNA gene phylotypes. (B) relative abundances of fungal ITS gene phylotypes. Bacterial phylum *Proteobacteria* and fungal phylum *Ascomycota* are subdivided into class. Shown fractions indicate the relative percentage to the total number of clones. For bacteria: Acido, *Acidobacteria*; Spiro, *Spirochaetes*; Tene, *Tenericutes*; Actino, *Actinobacteria*; Fibro, *Fibrobacteres*; Arma, *Armatimonadetes*; Bacter, *Bacteroidetes*; UN, Unclassified; Chlo, *Chloroflexi*; Cyano, *Cyanobacteria*; Elusi, *Elusimicrobia*; Firmi, *Firmicutes*; Gemma, *Gemmatimonadetes*; Nitro, *Nitrospirae*; Plancto, *Planctomycetes*; Alpha, *Alphaproteobacteria*; Delta, *Deltaproteobacteria*; Gamma, *Gammaproteobacteria*; Beta, *Betaproteobacteria*; CTM7, *Candidate division*; TM7 Verru, *Verrucomicrobia*.

### Rhizosphere soil

#### Soil sampling

Ten days later, rhizosphere soil samples were collected by shaking off from the roots in the air [28]. Soils from five plants per replicate were pooled to make a composite sample. After sieving (2 mm), subsamples of rhizosphere soils were monitored for soil pH, available N content, dehydrogenase activity and MBC estimation, whereas the other subsamples were stored at −70°C prior DNA extraction.

**Table 5:** LIBSHUFF statistical analysis for differences between bacterial 16S rRNA and fungal ITS gene sequences in clone libraries.

*p*-Coumaric acid concentration(µmol/g soil)	Bacterial community		Fungal community	
	0	0.5	0	0.5
0		0.0114, P<0.0001		0.033343, P<0.0001
0.5	0.0037, P = 0.0002		0.0367, P<0.0001	

Coverage (C) values of libraries are given in Δ*C*_AB_ (top diagonal) and Δ*C*_BA_ (lower diagonal) scores and significances are given in P values.

#### Soil phenol concentration

Soil phenols were extracted as previously described [32]. Briefly, 15 g soil was shaken in 100 ml of 2 M NaOH for 24 h (dark, 25°C). After centrifuged at 6000 g for 15 min, the supernatant was acidified to 2.5 with 5 M HCl, and extracted with ethyl acetate. The resulting extracts were evaporated to dryness at 40°C. The residue was dissolved in 5 ml of 80% methanol and filtered (0.22 µm). The methanol solution of soil extracts was analyzed with a Waters HPLC system (Waters, Milford, MA). The mobile phase was a mixture of 80% methanol and 20% water (0.8 ml/min). Detection was performed at 280 nm. Identification and quantification of *p*-coumaric acid were done by comparing the retention time and area with pure standard.

**Figure 6:**
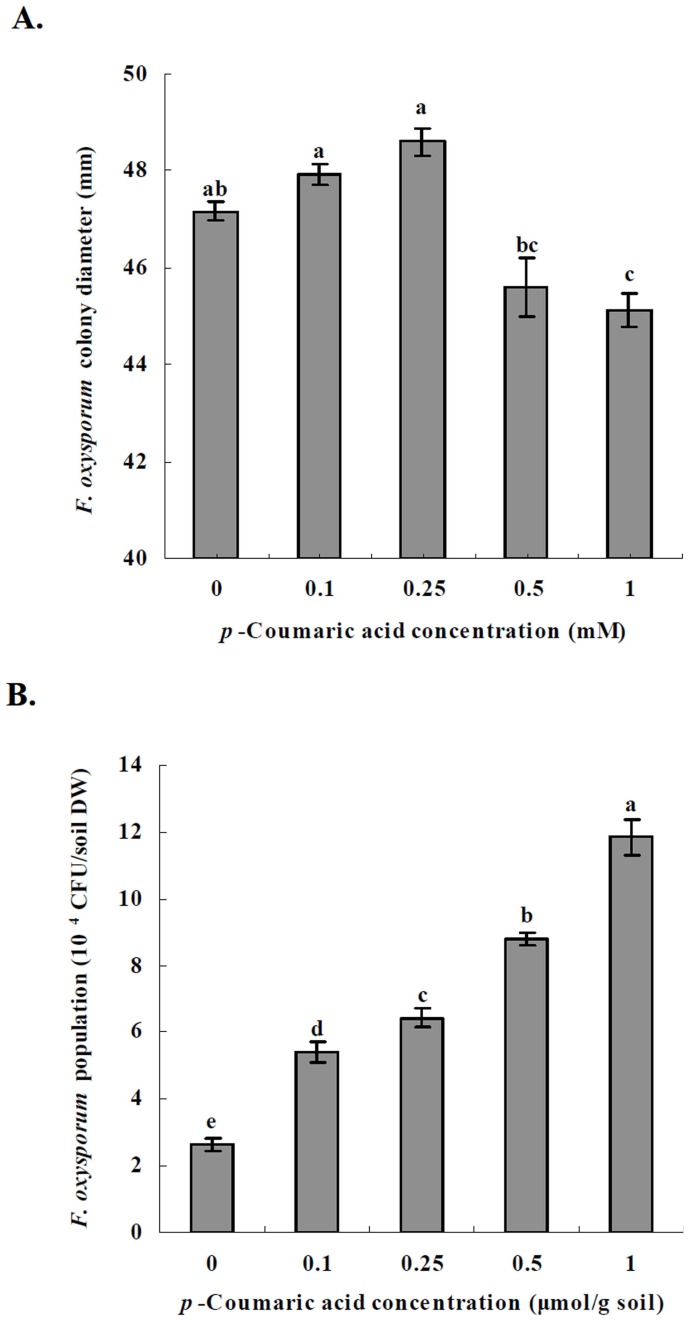
Effects of *p*-coumaric acid on *F. oxysporum* growth. (A) *F. oxysporum* colony diameter. (B) *F. oxysporum* population in the soil. Data are represented as the means of three independent replicates with standard error bars. *Different letters* indicate significant difference between treatments (P<0.05, Tukey’s HSD test). *CFU* colony forming unit.

#### Soil pH, available N, dehydrogenase activity and MBC

Soil pH was determined in water suspensions at a soil/water ratio of 1∶2.5 with a glass electrode. Available N (nitrate and ammonium) was extracted with 1 M KCl solution (1∶10, w/v) and analyzed with an autoanalyzer. Dehydrogenase activity was determined by the reduction of 2,3,5-triphenyltetrazolium chloride (TTC) [33]. Soil MBC was determined by the chloroform-fumigation-extraction method. An extractability factor of 0.38 was used to calculate MBC [34].

#### Real-time PCR

Bacterial and fungal community abundances were estimated by measuring bacterial 16S rRNA gene and fungal internal transcribed spacer (ITS) rRNA gene densities. Total soil DNA was extracted with an E.Z.N.A. Soil DNA Kit (Omega Bio-Tek, Inc., GA, USA). SYBR Green real-time PCR assays were conducted on an IQ5 real-time PCR system (Bio-Rad Lab, LA, USA) with 338F/518R [20] and ITS1F/ITS4 [35], [36] as primers, respectively. The PCR conditions were: 94°C for 5 min; 94°C for 45 s, 56°C for 45 s for bacterial 16S rRNA gene (57.5°C for 45 s for fungal ITS region), 72°C for 90 s, 30 cycles in total; a final elongation at 72°C for 10 min. Standard curves were created with a 10-fold dilution series of plasmids containing the 16S rRNA gene or ITS region. Soil DNA extracts were tested for inhibitory effect by diluting soil DNA extracts and by mixing a known amount of plasmid DNA with the DNA extracted from soil. In all cases, no inhibition was detected. Sterile water was used as a negative control to replace templates. All amplifications were replicated three times. The specificity of the products was confirmed by melting curve analysis and agarose gel electrophoresis. The threshold cycle (*Ct*) values obtained for each sample were compared with the standard curve to determine the initial copy number of the target gene.

#### PCR-DGGE

Bacterial and fungal community structures were estimated by the PCR-DGGE method. PCR amplification of partial bacterial 16S rRNA gene was performed with the GC-338F/518R primer [20]. A nested PCR protocol was used to amplify fungal ITS regions of the rRNA gene [35] with TS1F/ITS4 [35], [36] and GC-ITS1F/ITS2 [35] as primers for the first and second round of PCR amplifications, respectively. DGGE was performed using an 8% (w/v) acrylamide gel with 30–70% (bacteria) and 20–60% (fungi) denaturant gradient and run in a 1×TAE (Tris-acetate-EDTA) buffer for 14 h at 60°C and 80 V with a DCode universal mutation detection system (Bio-Rad Lab, LA, USA). After the electrophoresis, the gel was stained in 1∶3300 (v/v) GelRed (Biotium, CA, USA) nucleic acid staining solution for 20 min. DGGE profiles were photographed with an AlphaImager HP imaging system (Alpha Innotech Crop., CA, USA) under UV light.

#### Phylogenetic analysis

Bacterial and fungal community compositions were estimated by the clone library method. The bacterial 16S rRNA genes were amplified from soil DNA extracts with primers 27F/1492R and fungal ITS regions with primers ITS1F/ITS4 under previously described conditions [37], [38]. PCR products were cleaned using a TIANgel Midi Purification Kit (TIANGEN Biotech, Beijing, China) and then were inserted into pMD19-T vectors (Takara Biotech, Dalian, China) and transformed into JM109 competent cells (Takara Biotech, Dalian, China). Colonies were screened by *Eco*RI restriction endonuclease digestions [37] and were commercially sequenced using universal M13 forward and reverse primers.

### F. oxysporum Experiment

#### In vitro

A strain of *F. oxysporum* f.sp. *cucumerinum* Owen was isolated and identified from a *Fusarium* wilted cucumber grown in a greenhouse. The potato-dextrose-agar (PDA) medium was autoclaved at 120°C for 15 min [14]. When the medium was at 53°C, 2 ml of *p*-coumaric acid solution (filtered through 0.22 µm filter membranes) at pH 7.0 were added into 18 ml of PDA medium, and then thoroughly mixed and poured into Petri dishes. The final concentrations of *p*-coumaric acid were 0.1, 0.25, 0.5 or 1.0 mM, respectively. The medium added with the same amount of distilled water (pH 7.0) was used as the control. Afterwards, an agar plug (0.5 cm diameter) of *F. oxysporum* taken from a 7-d old PDA culture was inoculated on the center of these Petri dishes. There were five treatments (four concentrations of *p*-coumaric acid and one control) in total. We had three replicates for each treatment, and had five Petri dishes per replicate. After four days of incubation at 28°C in the dark, colony diameter was measured.

#### Microcosm

The soil used in the cucumber seedling experiment was used here. After sieving (2 mm), 50 g soil were inoculated with 10 ml of 10^4^ conidia/ml of *F. oxysporum* conidia suspension [27]. Then, different concentrations of *p*-coumaric acid (pH 7.0) were added to these *F. oxysporum*-inoculated soils every 48 h to achieve final concentrations of 0.1, 0.25, 0.5, 1.0 µmol/g soil DW. The soil treated with the same amount of distilled water (pH 7.0) was used as the control. Then, jars containing these treated soils were incubated at 28°C in the dark. The final soil water content was maintained at 30% of its water holding capacity. There were five treatments (four concentrations of *p*-coumaric acid and one control) in total. We had three replicates for each treatment, and had five jars per replicate. The colony forming unit (CFU) of culturable *F. oxysporum* population in the soil was measured 10 days after incubation with the plate counting method using the Komada medium [27].

### Statistical Analyses

Data were analyzed by analysis of variance (ANOVA) and mean comparison between treatments was performed based on the Tukey’s honestly significant difference (HSD) test at the 0.05 probability level with SAS 8.0 software. Banding patterns of the DGGE profiles and principal component analysis (PCA) were analyzed by the Quantity One software (version 4.5) and Canoco for Windows 4.5 software, respectively [39]. The DGGE evenness (*E*) and Shannon diversity (*H*) indices were calculated as described before [37].

Sequences from the clone libraries were edited manually to correct falsely identified bases and trimmed at both the 5′ and 3′ ends using the Chromas software. The presence of chimeras were screened using Bellerophon [40], and potential chimeras were excluded from further analysis. Mothur was used to assign sequences to operational taxonomic units (OTUs, 97% similarity) and calculate rarefaction curves, Chao 1 richness and Shannon diversity (*H*) indices [41]. LIBSHUFF in Mothur was used to test whether there were significant differences in bacterial or fungal community compositions between clone libraries. The Classifier function of the Ribosomal Database Project (http://rdp.cme.msu.edu/classifier/classifier.jsp) was used for broad taxonomic characterization of these sequences. The closest relatives of these sequences were determined at GenBank by the BLASTn algorithm. Phylogenetic analyses were conducted using MEGA (version 5.0) and the neighbour-joining tree was constructed using Kimura 2-parameter distance with 1000 replicates to produce Bootstrap values [42].

## Results

### Cucumber Radicle Elongation

*p*-Coumaric acid significantly inhibited cucumber radicle elongation (F_treatment_ = 92.40; df = 4,10; P<0.0001) and the inhibitory effects increased by increasing *p*-coumaric acid concentration (Fig. 1).

### Cucumber Seedling Growth

Generally, *p*-coumaric acid inhibited cucumber seedling growth and the magnitude of inhibition increased by increasing *p*-coumaric acid concentration (Table 1); *p*-coumaric acid significantly inhibited cucumber leaf area at concentrations equal or higher than 0.25 µmol/g soil, and significantly inhibited plant height and seedling dry weigh at 1.0 µmol/g soil (P<0.05).

### *p*-Coumaric Acid Retained in Soil

At the end of the cucumber seedling growth experiment, *p*-coumaric acid concentrations in cucumber-cultivated soils were significantly higher than in the soil before cucumber planting (P<0.05). Addition of *p*-coumaric acids increased *p*-coumaric acid concentration in soil, and the stimulatory effects increased by increasing concentration (Table 2).

### Soil pH and Available N

*p*-Coumaric acid had not significant effects on soil pH and available N content at concentrations equal or lower than 0.5 µmol/g soil, but significantly decreased soil available N content at 1.0 µmol/g soil (P<0.05) (Table 2).

### Soil Dehydrogenase Activity and MBC

*p*-Coumaric acid significantly increased soil dehydrogenase activity and MBC at all concentrations (P<0.05) (Table 2). Compared to the control soil, soil dehydrogenase activity and MBC were increased by 93.4–134.7% and 31.1–54.7%, respectively.

### Bacterial and Fungal Community Abundance

*p*-Coumaric acid significantly increased rhizosphere bacterial and fungal community abundances and decreased bacteria-to-fungi ratio (P<0.05) (Table 2). The soil treated with 0.25 µmol *p*-coumaric acid/g soil had the highest bacterial community abundance, while the soil treated with 0.5 µmol *p*-coumaric acid/g soil had the highest fungal community abundance.

### Bacterial and Fungal Community Structure

PCR-DGGE analyses showed that *p*-coumaric acid changed structures of rhizosphere bacterial (Fig. 2A) and fungal (Fig. 3A) communities. The DGGE banding patterns were similar in triplicate samples of each treatment for both bacterial (Fig. 2A) and fungal (Fig. 3A) communities, while were different among the soil before cucumber planting, the control soil (soil treated with water) and *p*-coumaric acid-treated soils. Number of visible bands, Shannon (*H*) and Evenness (*E*) indices of the bacterial community were smaller in *p*-coumaric acid-treated soils than in the control soil and the soil before cucumber planting (Table 3). These diversity indices decreased by increasing *p*-coumaric acid concentration. No definitive trend was observed in the indices of fungal community.

PCA analyses of bacterial (Fig. 2B) and fungal (Fig. 3B) DGGE banding patterns showed that *p*-coumaric acid-treated soils were different from the soil before cucumber planting and the control soil. For bacterial community, soils treated with 0.5 and 1.0 µmol/g soil *p*-coumaric acid grouped together, while soils treated with 0.1 and 0.25 µmol/g soil *p*-coumaric acid grouped together, indicating that high concentrations (0.5 and 1.0 µmol/g soil) and low concentrations (0.1 and 0.25 µmol/g soil) of *p*-coumaric acid had different influences on bacterial community structure.

### Bacterial and Fungal Community Composition

In total, we recovered 266 partial sequences of bacterial 16S rRNA and 284 fungal ITS gene sequences from the control soil and soil treated with 0.5 µmol *p*-coumaric acid/g soil (Table 4). The rarefaction analysis showed that bacterial 16S rRNA gene clone libraries were far from saturation with low coverages of 40% and 38% at 3% cut-off (Fig. 4 and Table 4); however, declines in the rate of OTUs were observed at 10% cut-off (data not shown), indicating that only the most dominant bacterial phyla were detected. For fungal ITS clone libraries, relatively higher coverages (90% and 86%) were observed and the rarefaction curves appeared to be closer to leveling off at 3% cut-off (Fig. 4 and Table 4). The Chao 1 richness index of the bacterial community was higher and the Shannon diversity index was smaller in *p*-coumaric acid-treated soils than in the control soil (Table 4).

Phylum-level characterization of the bacterial 16S rRNA gene clones showed that the bacterial clone libraries were largely composed of individuals representing the *Proteobacteria*, *Bacteroidetes* and *Acidobacteria* (Fig. 5A and Fig. S1). The relative abundances of *Bacteroidetes*, *Chloroflexi*, *Cyanobacteria*, *Planctomycetes* and *Verrucomicrobia* decreased while that of *Proteobacteria* and *Firmicutes* increased in the soil treated with 0.5 µmol *p*-coumaric acid/g soil. Within the *Proteobacteria* phylum, the relative abundances of *Betaproteobacteria* and *Gammaproteobacteria* classes increased while that of *Deltaproteobacteria* class decreased in the soil treated with 0.5 µmol *p*-coumaric acid/g soil. In the control soil, the *Deltaproteobacteria* class was dominated by *Myxococcales* (data not shown).

At the phylum-level, the fungal libraries were dominated by members of the *Ascomycota* and *Zygomycota* (Fig. 5B and Fig. S2). The relative abundance of *Zygomycota* increased in the soil treated with 0.5 µmol *p*-coumaric acid/g soil. With in the *Ascomycota* phylum, the relative abundance of *Sordariomycete* class increased and that of the *Pezizomycete* class decreased in the soil treated with 0.5 µmol *p*-coumaric acid/g soil.

The LIBSHUFF test revealed statistically significant differences in the bacterial and fungal community compositions between the libraries derived from the control soil and soil treated with 0.5 µmol *p*-coumaric acid/g soil (Table 5).

### *F. oxysporum* Mycelial Growth and Population

In the Petri dish experiment, *p*-coumaric acid slightly increased *F. oxysporum* colony diameter at 0.1 and 0.25 mM, but significantly decreased *F. oxysporum* colony diameter at 1.0 mM *in vitro* (F_treatment_ = 18.31; df = 4,10; P<0.0001) (Fig. 6A). In the microcosm experiment, all concentrations of *p*-coumaric acid significantly increased *F. oxysporum* population density in soil (F_treatment_ = 341.12; df = 4,10; P<0.0001) (Fig. 6B).

## Discussion

*p*-Coumaric acid concentrations in *p*-coumaric acid-treated soils were lower than the sum of the concentration in the control soil and the concentration applied, suggesting that part of this compound was consumed by microorganisms [43]. The physiological alterations caused by allelochemicals are concentration dependent, and for many phenols the range of bioactivity is between 0.1 and 1 mM [30]. Consistent with previous findings, detrimental effects of *p*-coumaric acid on cucumber radicle elongation and seedling growth were observed in this study. However, the concentration of phenols available to microorganisms may be different at different plant growth stages because phenols secreted by cucumber varied with plant growth stages [3]. Therefore, cucumber at different growth stages may respond differently to the same concentration of artificially applied phenols.

The inhibition of plant growth by plant litter or other types of organic matter may be due to microbial N immobilization or phytotoxicity [5], [44]. In this study, soil available N was only significantly decreased at the highest concentration (1.0 µmol *p*-coumaric acid/g soil). A previous study also showed that, in some cases, addition of N solution could not eliminate the phytotoxicity [45]. Therefore, N immobilization may not be responsible for the inhibition of cucumber seedling growth, at least at concentrations lower than 1.0 µmol *p*-coumaric acid/g soil. In fact, it was shown that phenols did not inhibit nitrification or ammonifiers in soil [46] and not retard ammonia oxidation by autotrophic microorganisms *in vitro* [47]. Special attention should be given to the long-term effects of phenols on soil N dynamics and microorganisms involved in N transformation.

In this study, soil dehydrogenase activity, an indicator of microbial activity, was increased by *p*-coumaric acid, which was consistent with the findings that phenol(s) can increase soil microbial activity [19], [48]. Soil MBC, and bacterial and fungal community abundance were also increased by *p*-coumaric acid, thus confirming what already observed that phenols can increase MBC [19] and the number of culturable microorganisms [17]. These increases were not surprising, considering that, phenols can be used as carbon sources by soil microorganisms [43].

Based on cultivation-dependent methods, previous studies showed that phenols affected the number of culturable soil microorganisms [17], [19]. With cultivation-independent methods (real-time PCR, PCR-DGGE and clone library analysis), we found that *p*-coumaric acid changed the abundance, structure and composition of soil microbial communities. Notably, *p*-coumaric acid stimulated and inhibited certain species of rhizosphere bacteria and fungi. The addition of phenols was shown to stimulate the growth of culturable soil microorganisms that are capable of degrading these compounds [17]. Members of the *Firmicutes* (e.g. *Bacillus licheniformis* [49]), *Gammaproteobacteria* (e.g. *Pseudomonas putida* and *Pseudomonas nitroreducens* [50], [51]), *Basidiomycota* (e.g. *Rhodotorula glutinis* [50]), *Betaproteobacteria* (e.g. *Alcaligenes faecalis* [52] and *Comamonas* [51]) and *Sordariomycetes* (e.g. *Phomopsis liquidambari* [53] and *Trichoderma harzianum* [29]) could degrade phenols. Thus, these increased bacteria and fungi may be responsible for degrading *p*-coumaric acid. The *Myxococcales* order contains a group of bacteria that predominantly feed on insoluble organic matter [54]. The *Verrucomicrobia* phylum is saccharolytic, oligotrophic and ubiquitous in soil [55]. The decreases in the relative abundances of *Myxococcales* and *Verrucomicrobia* implied that *p*-coumaric acid may decrease the soil microbial community’s ability to hydrolyze insoluble macromolecules.

PCR-DGGE analysis showed that the response of soil bacterial community structure to *p*-coumaric acid was concentration dependent, and PCR-DGGE and clone library analyses showed that *p*-coumaric acid decreased the diversity indices of bacterial community, however, these were not observed in fungal community. These indicated that rhizosphere soil bacterial and fungal communities responded differently to *p*-coumaric acid, confirming what already reported that soil bacterial and fungal communities responded differently to labile soil carbon (sugars, organic acids, and amino acids) inputs [10]. The different responses to these organic C additions to soil probably depend on the fact that bacteria use readily available organic compounds more quickly than fungi, whereas fungi are able to synthesize enzyme activities catalyzing the decomposition of more complex organic compounds [10].

The Petri dish experiment showed that *p*-coumaric acid inhibited *F. oxysporum* mycelial growth at 0.5 and 1.0 mM. *In vitro* inhibition of *p*-coumaric acid on *F. oxysporum* f.sp. *niveum* has been already demonstrated [14]. However, the microcosm experiment showed that *p*-coumaric acid stimulated *F. oxysporum* population in soil. These inconsistencies may be due to the different nutrient status of the environment in which *F. oxysporum* grew: nutrients are limited in soil while are in sufficient concentration in the artificial medium. Ye et al. [27] showed that phenols promoted the incidence of cucumber *Fusarium* wilt caused by *F. oxysporum*. Therefore, phenols may increase the population of *F. oxysporum* and the severity of *Fusarium* wilt under field conditions.

Phenols can affect plant root membrane permeability [56]; therefore, it is possible that *p*-coumaric acid could lead to the root leakage of organic compounds that can affect soil microbial communities. Effects of phenols (phenol 2,4-di-tert-butylphenol and vanillic acid) on bulk soil microorganism communities, such as stimulation of soil *Basidiomycota*, have been already observed [16], suggesting that changes in microbial communities observed in this study may be due to the added *p*-coumaric acid and the changed root exudation pattern.

Overall, our results validated the hypothesis that *p*-coumaric acid inhibited cucumber growth and changed rhizosphere soil microbial communities. Soil microorganisms are involved in many processes crucial to plant survival and performance [57]. Changes in soil microbial communities may affect functions performed by the microbial communities, which can influence plant growth [58], [59]. Thus, the bad performance of cucumber seedlings may be attributed to the changes in structure, composition, abundance, activity and size of soil microbial communities as affected by *p*-coumaric acids. Cucumber plant residues, which contain autotoxins, can accumulate in soil under continuously mono-cropped conditions. These autotoxins may affect soil microbial communities over time in the field. We have shown that, in the continuously mono-cropped cucumber system, the structure of rhizosphere fungal and *Fusarium* communities changed, whereas the abundances increased in the season that cucumber showed retarded growth [28]. A decline in the bacteria/fungi ratio was observed in ‘sick’ soils from continuous mono-cropping systems [26]. *p*-Coumaric acid increased the relative abundances of *Firmicutes* and *Proteobacteria* and decreased the relative abundance of *Cyanobacteria*, which was also found in soils under mono-cropped soybean [60]. The increase in the population of soil-borne pathogens (e.g. *F. oxysporum*) is thought to be responsible for the soil sickness [27]. Therefore, as an autotoxin, *p*-coumaric acid may account for the soil sickness of cucumber through changing soil microbial communities in natural ecosystems. Further researches should focus on gaining direct evidences that changes in soil microbial communities caused by accumulations of phenols are responsible for the bad performance of cucumber in the continuous mono-cropping system.

## Supporting Information

Figure S1 Neighbour-joining tree constructed from sequences from bacterial 16S rRNA gene clone libraries. (A) the control. (B) soil treated with *p*-coumaric acid (0.5 µmol/g soil). Trees display one representative clone per OTU (defined at the 97% similarity level) and their respective best match sequence with GenBank accession number. The phylogenetic distances of each sequence were calculated using the Kimura 2-parameter model and the tree was constructed using the neighbor-joining algorithm. Scale bars represent 5% changes. Bootstrap values (1 000 repetitions) above 50% are represented.
(PDF)

Figure S2 Neighbour-joining tree constructed from sequences from fungal ITS gene clone libraries. (A) the control. (B) soil treated with *p*-coumaric acid (0.5 µmol/g soil). Trees display one representative clone per OTU (defined at the 97% similarity level) and their respective best match sequence with GenBank accession number. The phylogenetic distances of each sequence were calculated using the Kimura 2-parameter model and the tree was constructed using the neighbor-joining algorithm. Scale bars represent 5% changes. Bootstrap values (1 000 repetitions) above 50% are represented.
(PDF)
