# Supplementary material for: p-Coumaric Acid Influenced Cucumber Rhizosphere Soil Microbial Communities and the Growth of Fusarium oxysporum f.sp. cucumerinum Owen
Source: PLoS One. 2012 Oct 30;7(10):e48288. doi: 10.1371/journal.pone.0048288 (PMC3484048; doi:10.1371/journal.pone.0048288)
Supplement: Figure S1 — Neighbour-joining tree constructed from sequences from bacterial 16S rRNA gene clone libraries. (A) the control. (B) soil treated with p-coumaric acid (0.5 µmol/g soil). Trees display one representative clone per OTU (defined at the 97% similarity level) and their respective best match sequence with GenBank accession number. The phylogenetic distances of each sequence were calculated using the Kimura 2-parameter model and the tree was constructed using the neighbor-joining algorithm. Scale bars represent 5% changes. Bootstrap values (1 000 repetitions) above 50% are represented. (PDF) [file pone.0048288.s001.pdf]

Figure S1

A.

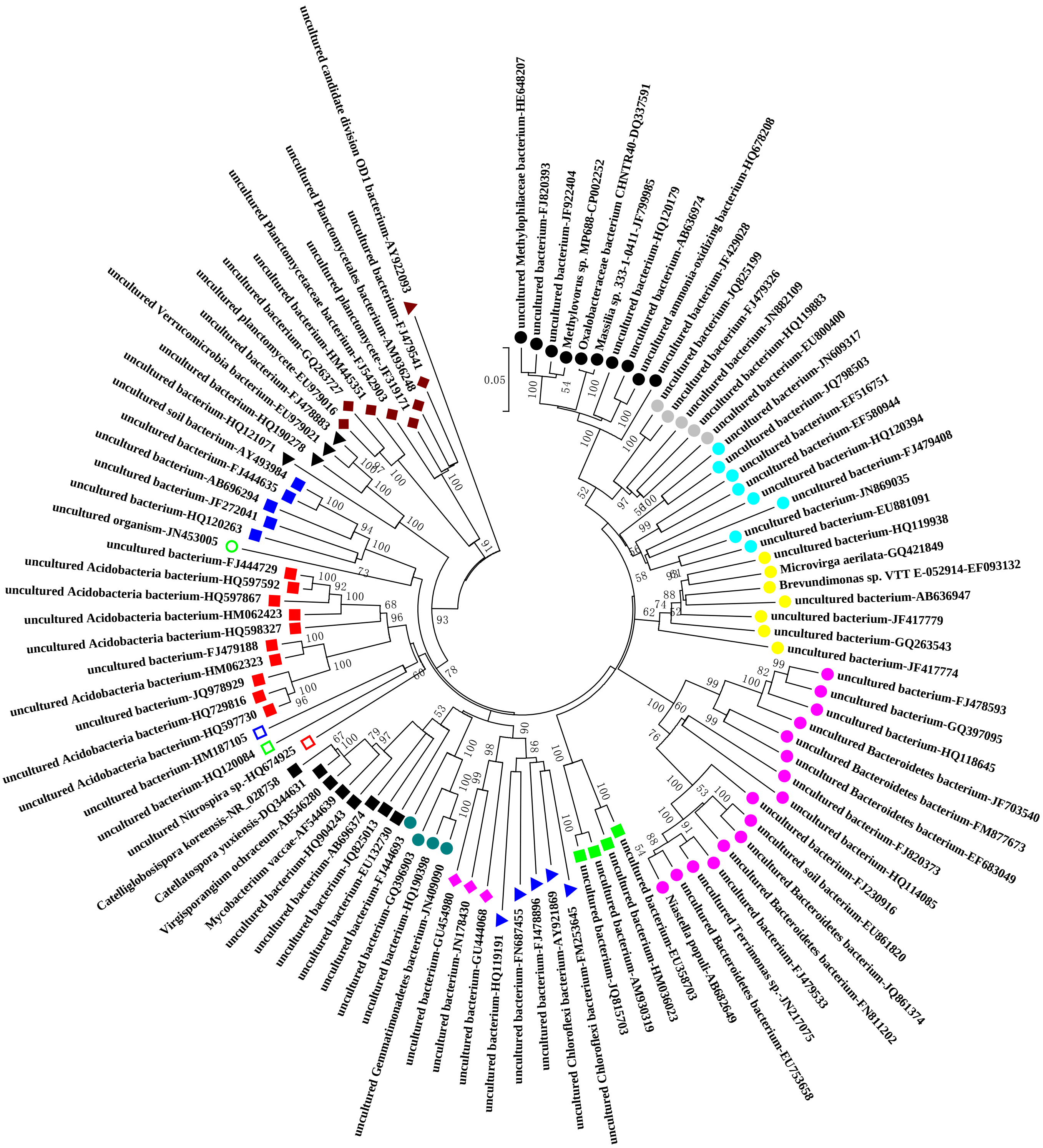
0.05
uncultured Methylophilaceae bacterium-HE648207
uncultured bacterium-FJ820393
uncultured bacterium-JF922404
Methylovorus sp. MP688-CP002252
Oxalobacteraceae bacterium CHNTR40-DQ337591
Massilia sp. 333-1-0411-JF799985
uncultured bacterium-HQ120179
uncultured bacterium-AB636974
uncultured ammonia-oxidizing bacterium-HQ678208
uncultured bacterium-JF429028
uncultured bacterium-JQ825199
uncultured bacterium-FJ479326
uncultured bacterium-JN882109
uncultured bacterium-HQ119883
uncultured bacterium-EU800400
uncultured bacterium-JN609317
uncultured bacterium-JQ798503
uncultured bacterium-EF516751
uncultured bacterium-EF580944
uncultured bacterium-HQ120394
uncultured bacterium-FJ479408
uncultured bacterium-JN869035
uncultured bacterium-EU881091
uncultured bacterium-HQ119938
Microvirga aerilata-GQ421849
Brevundimonas sp. VTT E-052914-EF093132
uncultured bacterium-AB636947
uncultured bacterium-JF417779
uncultured bacterium-GQ263543
uncultured bacterium-JF417774
uncultured bacterium-FJ478593
uncultured bacterium-GQ397095
uncultured bacterium-HQ118645
uncultured Bacteroidetes bacterium-JF703540
uncultured Bacteroidetes bacterium-FM877673
uncultured Bacteroidetes bacterium-EF683049
uncultured bacterium-FJ820373
uncultured bacterium-HQ114085
uncultured bacterium-FJ230916
uncultured soil bacterium-EU861820
uncultured Bacteroidetes bacterium-JQ861374
uncultured Bacteroidetes bacterium-FN811202
uncultured bacterium-FJ479533
uncultured Terrimonas sp.-JN217075
uncultured Bacteroidetes bacterium-EU753658
Niastella populi-AB682649
uncultured bacterium-EU358703
uncultured bacterium-HM036023
uncultured bacterium-AM930319
uncultured bacterium-JQ815703
uncultured Chloroflexi bacterium
uncultured Chloroflexi bacterium-FM253645
uncultured bacterium-AY921869
uncultured bacterium-FJ478896
uncultured bacterium-FN687455
uncultured bacterium-HQ119191
uncultured bacterium-GU444068
uncultured bacterium-JN178430
uncultured bacterium-GU454980
uncultured Gemmatimonadetes bacterium-JN409090
uncultured bacterium-HQ190398
uncultured bacterium-GQ396903
uncultured bacterium-FJ444693
uncultured bacterium-EU132730
uncultured bacterium-JQ825013
uncultured bacterium-AB696374
uncultured bacterium-HQ904243
Mycobacterium vaccae-AF544639
Virgisporangium ochraceum-AB546280
Catellatospora yuxiensis-DQ344631
Catelliglobosispora koreensis-NR_028758
uncultured Nitrospira sp.-HQ674925
uncultured bacterium-HQ120084
uncultured bacterium-HM187105
uncultured Acidobacteria bacterium-HQ597730
uncultured Acidobacteria bacterium-HQ729816
uncultured bacterium-JQ978929
uncultured Acidobacteria bacterium-HM062323
uncultured bacterium-FJ479188
uncultured Acidobacteria bacterium-HQ598327
uncultured Acidobacteria bacterium-HM062423
uncultured Acidobacteria bacterium-HQ597867
uncultured Acidobacteria bacterium-HQ597592
uncultured bacterium-FJ444729
uncultured organism-JN453005
uncultured bacterium-HQ120263
uncultured bacterium-JF272041
uncultured bacterium-AB696294
uncultured bacterium-FJ444635
uncultured soil bacterium-AY493984
uncultured bacterium-HQ121071
uncultured bacterium-HQ190278
uncultured Verrucomicrobia bacterium-EU979021
uncultured bacterium-FJ478883
uncultured planctomycete-EU979016
uncultured bacterium-GQ263727
uncultured bacterium-HM445351
uncultured Planctomycetaceae bacterium-FJ542903
uncultured planctomycete-JF319171
uncultured Planctomycetales bacterium-AM936248
uncultured bacterium-FJ479541
uncultured candidate division OD1 bacterium-AY922093

B.

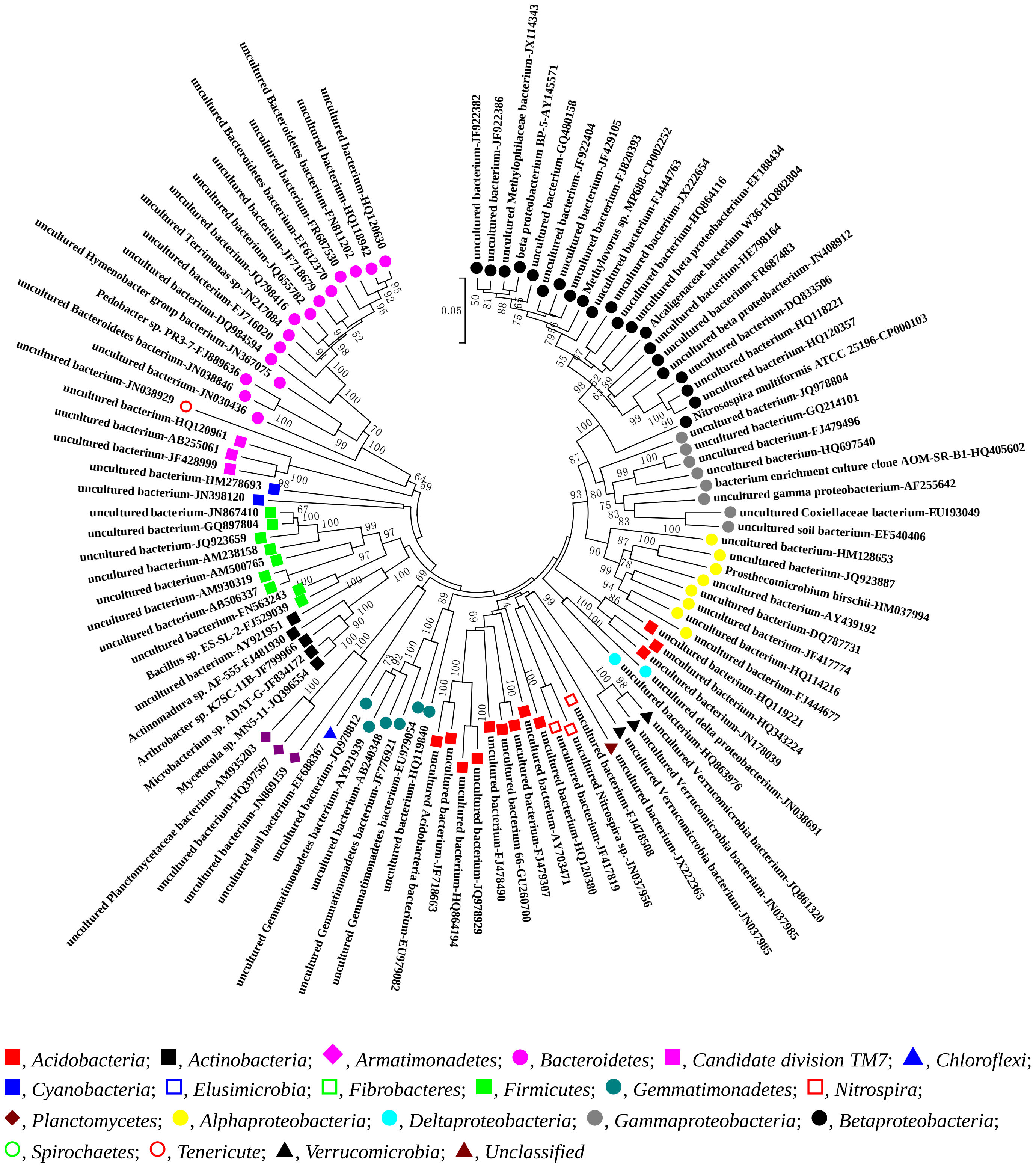
uncultured bacterium-JF922382
uncultured bacterium-JF922386
uncultured Methylophilaceae bacterium-JX114343
beta proteobacterium BP-5-AY145571
uncultured bacterium-GQ480158
uncultured bacterium-JF922404
uncultured bacterium-JF429105
uncultured bacterium-FJ820393
Methylovorus sp. MP688-CP002252
uncultured bacterium-FJ444763
uncultured bacterium-JX222654
uncultured bacterium-HQ864116
uncultured beta proteobacterium-EF188434
Alcaligenaceae bacterium W36-HQ882804
uncultured bacterium-HE798164
uncultured bacterium-FR687483
uncultured beta proteobacterium-JN408912
uncultured bacterium-DQ833506
uncultured bacterium-HQ118221
uncultured bacterium-HQ120357
Nitrosospira multiformis ATCC 25196-CP000103
uncultured bacterium-JQ978804
uncultured bacterium-GQ214101
uncultured bacterium-FJ479496
uncultured bacterium-HQ697540
bacterium enrichment culture clone AOM-SR-B1-HQ405602
uncultured gamma proteobacterium-AF255642
uncultured Coxiellaceae bacterium-EU193049
uncultured soil bacterium-EF540406
uncultured bacterium-HM128653
uncultured bacterium-JQ923887
Prosthecomicrobium hirschii-HM037994
uncultured bacterium-AY439192
uncultured bacterium-DQ787731
uncultured bacterium-JF417774
uncultured bacterium-HQ114216
uncultured bacterium-FJ444677
uncultured bacterium-HQ119221
uncultured bacterium-HQ343224
uncultured bacterium-JN178039
uncultured delta proteobacterium-JN038691
uncultured bacterium-HQ863976
uncultured Verrucomicrobia bacterium-JQ861320
uncultured Verrucomicrobia bacterium-JN037985
uncultured Verrucomicrobia bacterium-JN037985
uncultured bacterium-JX222365
uncultured Nitrospira sp.-JN037956
uncultured bacterium-FJ478508
uncultured bacterium-JF417819
uncultured bacterium-HQ120380
uncultured bacterium-AY703471
uncultured bacterium-FJ479307
uncultured bacterium 66-GU260700
uncultured bacterium-FJ478490
uncultured bacterium-JQ978929
uncultured bacterium-HQ864194
uncultured bacterium-JF718663
uncultured Acidobacteria bacterium-EU979082
uncultured bacterium-HQ119840
uncultured Gemmatimonadetes bacterium-EU979054
uncultured Gemmatimonadetes bacterium-JF776921
uncultured Gemmatimonadetes bacterium-AB240348
uncultured bacterium-AY921939
uncultured bacterium-JQ978812
uncultured soil bacterium-EF688367
uncultured bacterium-JN869159
uncultured bacterium-HQ397567
uncultured Planctomycetaceae bacterium-AM935203
Mycetocola sp. MN5-11-JQ396554
Microbacterium sp. ADAT-G-JF834172
Arthrobacter sp. K7SC-11B-JF799966
Actinomadura sp. AF-555-FJ481930
uncultured bacterium-AY921951
Bacillus sp. ES-SL-2-FJ529039
uncultured bacterium-FN563243
uncultured bacterium-AB506337
uncultured bacterium-AM930319
uncultured bacterium-AM500765
uncultured bacterium-AM238158
uncultured bacterium-JQ923659
uncultured bacterium-GQ897804
uncultured bacterium-JN867410
uncultured bacterium-JN398120
uncultured bacterium-HM278693
uncultured bacterium-JF428999
uncultured bacterium-AB255061
uncultured bacterium-HQ120961
uncultured bacterium-JN038929
uncultured Bacteroidetes bacterium-JN030436
Pedobacter sp. PR3-7-FJ889636
uncultured Hymenobacter group bacterium-JN038846
uncultured bacterium-JN367075
uncultured bacterium-DQ984594
uncultured bacterium-FJ716020
uncultured Terrimonas sp.-JN217084
uncultured bacterium-JQ798416
uncultured bacterium-JQ655782
uncultured Bacteroidetes bacterium-FJ718679
uncultured bacterium-EF612370
uncultured bacterium-FR687530
uncultured Bacteroidetes bacterium-FN811202
uncultured bacterium-HQ118942
uncultured bacterium-HQ120630
0.05
, Acidobacteria; , Actinobacteria; , Armatimonadetes; , Bacteroidetes; , Candidate division TM7; , Chloroflexi;
, Cyanobacteria; , Elusimicrobia; , Fibrobacteres; , Firmicutes; , Gemmatimonadetes; , Nitrospira;
, Planctomycetes; , Alphaproteobacteria; , Deltaproteobacteria; , Gammaproteobacteria; , Betaproteobacteria;
, Spirochaetes; , Tenericute; , Verrucomicrobia; , Unclassified
