# Supplementary material for: p-Coumaric Acid Influenced Cucumber Rhizosphere Soil Microbial Communities and the Growth of Fusarium oxysporum f.sp. cucumerinum Owen
Source: PLoS One. 2012 Oct 30;7(10):e48288. doi: 10.1371/journal.pone.0048288 (PMC3484048; doi:10.1371/journal.pone.0048288)
Supplement: Figure S2 — Neighbour-joining tree constructed from sequences from fungal ITS gene clone libraries. (A) the control. (B) soil treated with p-coumaric acid (0.5 µmol/g soil). Trees display one representative clone per OTU (defined at the 97% similarity level) and their respective best match sequence with GenBank accession number. The phylogenetic distances of each sequence were calculated using the Kimura 2-parameter model and the tree was constructed using the neighbor-joining algorithm. Scale bars represent 5% changes. Bootstrap values (1 000 repetitions) above 50% are represented. (PDF) [file pone.0048288.s002.pdf]

Figure S2

A.

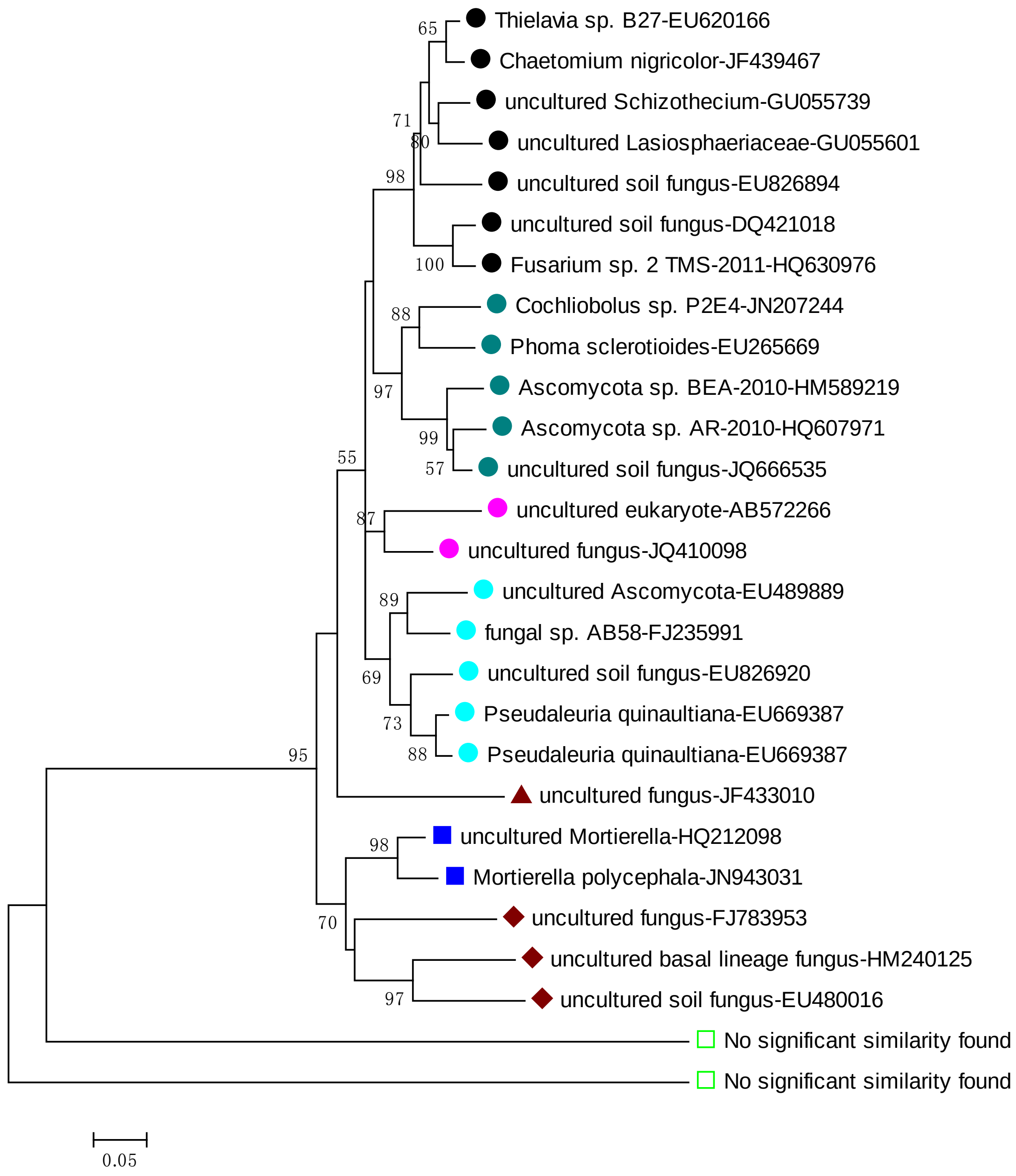

Thielavia sp. B27-EU620166
Chaetomium nigricolor-JF439467
uncultured Schizothecium-GU055739
uncultured Lasiosphaeriaceae-GU055601
uncultured soil fungus-EU826894
uncultured soil fungus-DQ421018
Fusarium sp. 2 TMS-2011-HQ630976
Cochliobolus sp. P2E4-JN207244
Phoma sclerotioides-EU265669
Ascomycota sp. BEA-2010-HM589219
Ascomycota sp. AR-2010-HQ607971
uncultured soil fungus-JQ666535
uncultured eukaryote-AB572266
uncultured fungus-JQ410098
uncultured Ascomycota-EU489889
fungal sp. AB58-FJ235991
uncultured soil fungus-EU826920
Pseudaleuria quinaultiana-EU669387
Pseudaleuria quinaultiana-EU669387
uncultured fungus-JF433010
uncultured Mortierella-HQ212098
Mortierella polycephala-JN943031
uncultured fungus-FJ783953
uncultured basal lineage fungus-HM240125
uncultured soil fungus-EU480016
No significant similarity found
No significant similarity found
65
71
80
98
100
88
97
99
57
55
87
89
69
73
88
95
98
70
97
0.05

B.

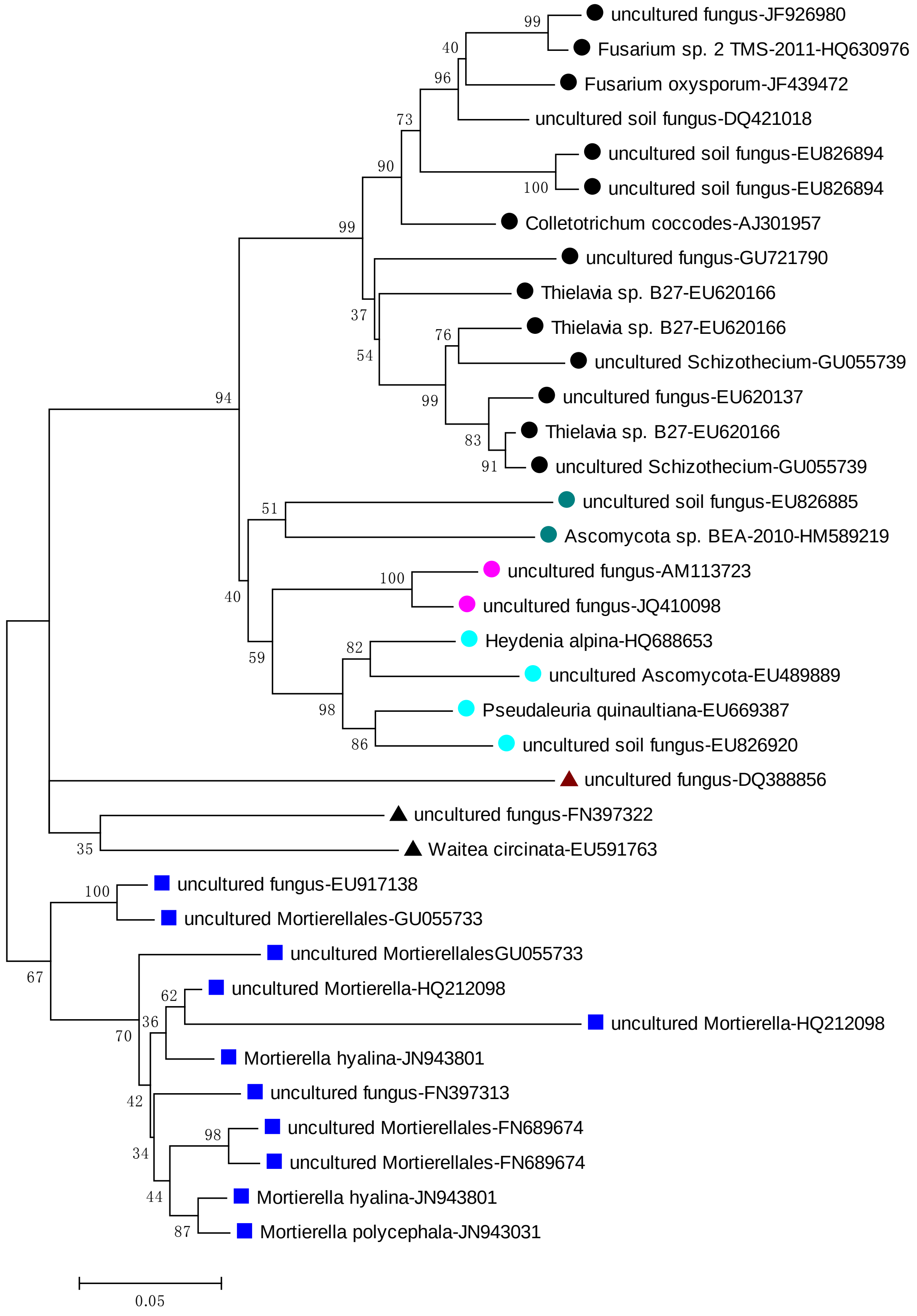

●, *Sordariomycete*; ●, *Dothideomycete*; ●, *Leotiomycete*; ●, *Pezizomycete*; ▲, *Unclassified*;
▲, *Basidiomycota*; ◆, *Chytridiomycota*; ■, *Zygomycota*; □, Unknown
